# Mean-Fields and Neural Masses

**DOI:** 10.1371/journal.pcbi.1000081

**Published:** 2008-08-29

**Authors:** Karl Friston

**Affiliations:** University College London, London, United Kingdom

This issue's front section features a special Review: **The Dynamic Brain: From Spiking Neurons to Neural Masses and Cortical Fields**. I was asked to write this Editorial on behalf of the editors and coauthors of the article, as an introduction— an introduction that we hope will serve as an aperitif for the article itself. The Review was solicited by Rolf Kötter (*PLoS Computational Biology* Reviews Editor) at one of the annual Brain Connectivity Workshops (http://www.hirnforschung.net/bcw), which we cofounded many years ago. These are highly interactive, discussion-orientated meetings that focus on the latest advances in modelling functional integration and coupling in the brain. They naturally attract a broad range of computational neuroscientists, neurophysiologists, neuroanatomists, and cognitive scientists. That year, we seemed drawn to computational neurobiology and the models of neuronal dynamics that were being used to understand interactions in the brain.

After a couple of days, it became clear that we were all using exactly the same rhetoric in reference to completely different things. Terms such as mean-field approximations, mass-action, neural-mass models, neural-field models, density-dynamics, etc., were used with exuberance but did not really help us to communicate. As a result, Rolf challenged us to define and synthesise these perspectives in a coherent and pragmatic way; the response to that challenge is the article in this issue of *PLoS Computational Biology* by Deco, Jirsa, Robinson, Breakspear, and Friston, which took more than two years to prepare. The difficulties became evident quickly when we started to integrate our respective contributions. These were far from coherent and disclosed some fundamental differences in the perspectives adopted on modelling distributed brain activity at a statistical level. These differences ranged from the semantic (e.g., what does “mass” mean in neural-mass models) to the sublime (e.g., some of us clearly found it difficult, if not heartbreaking, to give up our favourite variables to ensure notational consistency). This, in part, reflects the fact that the coauthors are all autonomous and senior scientists in their own fields, working on different continents, and emerging from very distinct intellectual backgrounds. Even the way in which these models were being used differed markedly among the contributors. After numerous iterations and cross-revision, the end product started to attain a degree of coherence (although some might argue there is still room for improvement).

The basic issue our synthesis tries to address is how different models, used to simulate and predict observed brain dynamics, can be traced back to their common fundaments. We then try to illustrate the diversity of applications that can be entertained with these models. The basis of these models rests on modelling, not on the behaviour of individual nerve cells or neurons, but on the probability density over ensembles or populations of similar neurons. The Fokker-Planck formalism becomes central here and can be harnessed using neuronal models that are cast in terms of differential equations, with or without discrete behaviours (e.g., neuronal spiking or firing). From the density dynamics afforded by the Fokker-Planck equation, we then pursue various simplifications and special cases. An important example is when the density becomes a point-mass over the expected states of a population. These are referred to as neural-mass models and predominate in the computational neuroscience literature. A key generalisation of these neural-mass models is to neural-field models, where the location of the mass or expected state of a population becomes a function of both time and position on the brain's cortical surface or subcortical structures. These models generate all sorts of interesting and neuronally plausible patterns and self-organising phenomena, which can be inferred through invasive or non-invasive electrophysiological recordings of real brains.

The applications of these models are essentially twofold; some authors use them to understand the basic principles of neuronal dynamics and implicit computations; for example, understanding dynamics in terms of nonlinear mechanisms such as bifurcations, understanding perceptual categorisation in terms of multistability, or identifying the domains of parameter–space that support commonly observed spatiotemporal patterns of activity. Other authors use these models as forward or generative models, whose parameters can be optimised to reproduce an observed dataset. This is known as model inversion and allows one to estimate important biophysical constants and parameters from empirical data. In this context, one can also explore model spaces and use data to adjudicate among various neural-mass or field models. We have chosen a few key examples that highlight the necessary role of density-dynamics and mean-field approximations in computational neuroscience.

Participating in this Review has been an enormously enlightening experience; at the same time it was exhausting and something I will think twice about before engaging in again. This is not meant to be a brief orientation to the field but more an attempt to provide a reference framework for people to understand their own contributions, in relation to others. It is a Review that I look forward to giving to my students; although I suspect they will take more than a weekend to digest it.

